# Keyword Index for Volume 104

**DOI:** 10.1038/bjc.2011.212

**Published:** 2011-06-07

^1^H-MRS 1854 4T1 cell 128

5-aminolevulinic acid 798 5-fluorouracil 1067, 1459 5-FU 1691

8q24.3 376

*α*-TEA 101 actin cytoskeleton 781 activated Kras 1038 activin 1303 acute lymphoblastic leukaemia 12, 746 acute myeloid leukaemia 1482 adenocarcinoma 700, 1296 adenoma–carcinoma sequence 1426 adhesion 345 adipose tissue 441 adjuvant chemotherapy 559 adjuvant treatment 899 adoptive transfer 948 adverse events 899 AGC diagnosis 353 age 564 aggressive fibromatosis 1452 Akt 101, 1755 AKT/PKB and S6K 1116 albumin 726 alcohol 537, 1487 ALKBH3 700 allele-specific expression 735 allelic imbalance 735 ambulatory care 1377 analgesics 532, 1704 anastrozole 1059 angiogenesis 83, 166, 635, 819, 1144, 1185, 1628, 1822, 1877 angiopoietin-2 51 anthracycline resistance 1071 anticancer agent 448, 635 anticancer immunotherapy 300 anticoagulation 413 antifolates 272 antimetabolites 272 anti-metastatic 635 anti-oestrogen therapy 1828 anxiety 255, 578 AP-1 1920 APC 1452 apoptosis 91, 235, 437, 781, 1151, 1278, 1459, 1594 apoptosis and multiple myeloma 957 ARHGEF16 324 ARMS 982 aromatase inhibitors 1059, 1558 array-CGH 361, 376 Asian population 871 ASPM 1602 astrocytic tumours 968 astrocytoma 1747 attenuated total reflection Fourier-transform infrared spectroscopy 790 attitudes 1535 autoantibodies 1896 auxological 746 avoidable deaths 1836 AZD1152 769

*β*-1 integrin 1587 *β*-catenin 1452 Bag-1 1459 balancing baseline covariates 1711 basal 120 Bcl-2. 120 bevacizumab 413, 599, 1079, 1262, 1270 bim 281 biomarkers 719, 1144, 1262, 1313, 1325, 1602, 1755, 1896 bladder cancer 376, 808, 1135, 1869 BMP-7 714 body mass index 746, 871, 921, 1207 bone metastasis 505, 613 *BRAF* 392, 856 BRAF^V600E^ 392 brain 1877 brain metastasis 1816 brain tumour stem cell 448 *BRCA1* 316, 903, 1349, 1356, 1384 *BRCA2* 1356, 1384 breast cancer 6, 101, 128, 332, 338, 419, 524, 559, 564, 571, 578, 871, 899, 910, 1059, 1214, 1342, 1356, 1384, 1551, 1558, 1628, 1656, 1680, 1716, 1730, 1828 Breast Cancer Index 1762 breast cancer mortality 910 breast cancer stem cells 1564 breast conservation 1551 breast density 871 breast neoplasms 60, 520 breath 1649 BRG1 146 brick dust 1797 BRM 146

CA125 863, 989 cachexia 441 CA-IX 353 cancer awareness 1836 cancer chemotherapy 448 cancer management 1246, 1535 cancer progression 841 cancer registry 564 cancer risk 1493 cancer stem cells 1410, 1730 cancer-associated inflammation 1288 cancer-predisposing gene 735 capecitabine 43, 1067, 1071 capture-recapture 1227 carcinogenesis 208, 229 case–control studies 175, 188, 875, 910, 1207, 1482 caspase-8 281, 1278 castrate-resistant prostate cancer 613, 1920 cathepsin B 957 CD9 496 CD9P-1 496 CDK inhibitor 1862 CDX2-positive circulating tumour cells 1000 celecoxib 1822 cell cycle 235, 1523 cell line 968 cell migration 345 CellSearch system 1472 cell-to-cell interaction 505 central venous blood 1472 centrosome 1523 c-erb-b2 1739 cerebrovascular disease 899 cervical cancer 361, 685, 971 cervical cancer precursors 110 cervical cancer prevention 248 cervical intraepithelial neoplasia 685 cervix 353, 1500 cetuximab 427, 488, 1691, 1786 chemoradiotherapy 265, 361 chemoresistance 19, 707, 1730 chemoresistant breast cancer 1401 chemotherapy 407, 750, 856, 1670, 1810, 1840 Chernobyl nuclear accident 181 childhood 746 childhood cancer 214, 1227 childhood leukaemia 12, 1227 China 1511 Chinese 369 cholangiocarcinoma 24, 587, 1313 choline and phosphocholine release, MARCKS 673 chromosomal instability 1724 chronic disease 399 cilengitide 1691 CIP2A 1890 circulating endothelial progenitor cells 1144 circulating tumour cells 1178, 1472, 1913 cisplatin 265, 593, 1071, 1098, 1126, 1278, 1691 citation network analysis 1085 clear cell 1151 clinical trials 1529, 1535 cohort 419, 537, 1196, 1500, 1511 colon cancer 33, 769, 882, 1178, 1202, 1426 colon polyp 882 colorectal cancer 6, 43, 60, 146, 188, 193, 369, 413, 480, 488, 856, 893, 934, 1000, 1007, 1013, 1020, 1079, 1085, 1262, 1270, 1288, 1434, 1619, 1697, 1770, 1779, 1786, 1791, 1906 colorectal cancer predisposition 735 colorectal cancer survival 763 colposcopy 255 combination chemotherapy 272 combinatorial therapy 399 co-morbidity 60 complete case analysis 693 compliance 1558 concordance 1020 contralateral breast cancer 1384 cost effectiveness 1377 coumarin 91 COX-2 763, 819 coxsackie adenovirus receptor 1426 C-reactive protein 51, 726 CRPC 605, 620 CTC 1913 CTL 300 cucurbitacin IIa 781 cutaneous melanoma 982 CXCL1 480 CXCL8 480 CXCL9 469 CXCR1 480 CXCR2 480 CXCR4 1805 cyclin D1 146 CYP2A6 1126 CYP450R 1098 cytokeratin 1434, 18 719 cytology 1325 cytotoxic CD8^+^ T cells 948

dacarbazine 750, 1816 DCIS 120 DcR1 1313 DDS 593 delayed diagnosis 934, 1249, 1836 delayed-type hypersensitivity 433 deleted in oesophageal cancer 1 (DEC1) 841 deoxyribose 1185 depression 255 deprivation 1505, 1791 desmoid tumour 37 desmoid-type fibromatosis 37 dexamethasone 605, 620 diagnosis 110, 1325, 1418 diagnostic imaging 1482 diagnostic marker 893 dietary patterns 524, 1493 diethylstilbestrol 605, 620 differentiation therapy 229 disseminated tumour cells 1434 distant metastasis 1724 distress 255, 419 disulfiram 1564 DNA damage 1869 DNA methylation 1013, 1313 DNA microarray 1027 DNA repair 653, 1349 DNA-PK 1724 docetaxel 265, 613 dormancy 1628 doxorubicin 437, 559, 957 DR5 281, 1278 drug resistance 272 DSC3 1013 dynamic allocation 1711 dysplasia 460

early death 1544 early diagnosis 1836 early presentation 1836 early-onset breast cancer 903 early-stage breast cancer 1913 education 520 *EGFR* 338, 488, 968, 989, 1135 EGFR family 1241 EGFR promoter methylation 1786 elderly 1393 EMT 989 end of life 1704 endocrine therapy 1393 endometrial cancer 790, 921, 1418, 1207, 1505, 1611, 1836 endometrium 790 endothelial monolayer breakdown 469 enrichment 1913 EpCAM 1434 epidemiology 532, 763, 1202, 1493 epidermal development 229 epidermal growth factor receptor 1575 epigenetic 1500 epigenome 554 epirubicin 613 epithelial ovarian cancer 1602 epithelial–mesenchymal transition 1160 EPR effect 593 Epstein-Barr virus 332 ER 1393 ERBB2 1135, 1739 ErbB2/HER2 1716 ERBB3 1135, 1241 ERCC1 1126 ERK 101 Ewing tumour 948 exemestane 1059 exon array 971 expression profiling 971 extracapsular spread 850

factor analysis 524 factor VII 1401 faecal occult blood testing 1779 faeces 1770 familial adenomatous polyposis 37 febrile neutropenia 407 ferrochelatase 798 fever 1377 FFPE 971 FGFR1 75 FGFR3 75 fibreglass 1797 figitumumab 68 FIH-1 1151 first generation immigrants 1193 FISH 1372 FKBP5 19 FLT3 ligand 719 fluid 1487 fluorescence 798 fluorescence *in situ* hybridisation 850 folate transporters 272 FOLFOX-4 427 fruit 6 fugetaxis 469 FXR 1027

G1/S checkpoint 1862 gall bladder cancer 587 GalNAc-T3 1882 GalNAc-T6 1882 gastric cancer 166, 714, 1126, 1372, 1511, 1840 gastrointestinal cancer 441 gastrointestinal stromal tumour 1686 GC–MS 1649 GDF15 1619 gemcitabine 769, 1071 gene expression profiling 545, 1762 genetic 369, 1049 GLI1 1575 glioblastoma 545, 968, 1365, 1805, 1810 glioma 193, 545, 653, 798 glutathione 437 GPRD 193 growth factor 1641 GTD 1665 GUT-70 91

haemorrhage 413 hand pattern 175 HCG 1665 HDAC 1828 HDAC inhibitors 957, 1828 HE4 863, 1418 head and neck cancer 830, 1641, 1649, 1896 head and neck squamous cell carcinoma 1319 heat shock protein 643 heat shock protein-70 1459 hedgehog 1575 *Helicobacter pylori* 198, 1511 heparan sulfate 635 heparanase 635 hepatectomy 1007 hepatic metastasectomy 1085 hepatitis B reactivation 559 hepatocellular carcinoma 24, 1303 HER2 332, 1372, 1739, 1920 *HER2* amplification 1342 HER2 positive 1675 HER2/neu 1739 herbal medicines 927 herpesviruses 433 heterogeneity 361, 1739 H-HPV 353 HIF-1*α* 166, 1151 HIF-2*α* 1151 Hippo 24 histological type 198 histology 714, 971, 1505 histone deacetylase 756, 1828 history of medicine 1085 HLA-transgenic mouse 300 Hodgkin and non-Hodgkin lymphoma 719 Hoechst 33342 dye 1410 hormone therapy 763 HPV 830, 886, 915, 1334, 1896 HPV prevalence, unvaccinated adolescents, Scotland 1221 HPV test 248 HPV16 E6 324 Hsp90 91, 629 human apurinic/apyrimidinic endonuclease 1 (APE1) 653 human immunodeficiency virus (HIV) 433 HuR 819 hyperglycaemia 345 hyperinsulinemia 345 hyperplasia 460 hypertension 599 hyperthyroidism 1665 hypothyroidism 241 hypoxia 166, 1319

IBC 1575 *IDH1* 968 IGF-1 68 IGFBP-2 1587 IGF-independent 1587 IGF-IR 68 IL-1 128 imatinib 1686 immunohistochemistry 110, 1372, 1440, 1459, 1594 immunophilins 19 immunoRNase 1716 immunosuppression 1334 immunotherapy 948, 1106, 1256, 1716 incidence 178, 1505, 1791 industry 208 infants 532 infection 407 influenza 1670 infrared spectroscopy 790 inhibin 1303 iNOS 83 in-patients 1377 insulin resistance 51 integrin 33 interleukin-2 1256 interval cancer 571 intrinsic subtypes 120 invasion 155, 1038 invasive phenotype 664 iodine deficiency 181 iodine radioisotopes 181 ionising radiation 1049, 1482 irradiation 1805 irritable bowel syndrome 1202 IRS-1 101 Italy 433

JAK2/STAT3 781

Kaposi sarcoma 433 KIF20A/RAB6KIFL/MKlp2 300 kinase inhibitor 1116 *KRAS* 856 *KRAS* mutation 1020

laser-capture microdissection 464 late effects 746 latent membrane protein 1 1160 lead time bias 1680 leptin 128 letrozole 1059 leukaemia 12, 532 ligand-targeted photodynamic therapy 1401 lipolysis 441 liver cancer 24, 235 liver fluke 1313 liver function tests 726 liver metastases 1020, 1079 LLETZ 255 low-dose chemotherapy 1822 low-grade glioma 1747, 1854 luminex 1896 lung cancer 6, 208, 496, 700, 1649, 1704 LY6K 376 lymph node 850 lymph node metastasis 1027

malignancy 1478 mammalian reovirus 290 mammography 871, 1214 mammography screening 571 management 37, 564, 1810 mantle cell lymphoma 91 MAP kinase pathway 1920 MAPK pathway 1564 MARIBS 578 marital status 1249 marker protein patterns 110 mastectomy 1551 meat 1196 Mediterranean diet 1493 MEK/ERK 982 melanoma 155, 392, 643, 653, 750 melanoma progression 982, 1816 meningioma 1049 merkel cell carcinoma 178 merkel cell polyomavirus 178 mesothelioma 1325 meta-analysis 1196, 1440 metastasis 235, 629, 1007, 1038, 1160, 1270, 1877, 1882 metastatic breast cancer 1071, 1472, 1675 metastatic colorectal cancer 1848 metastatic renal cell carcinoma 1144 methotrexate 272 methylation 1500, 1770, 1906 MIC-1 1619 microarrays 545 microcephalin 1602 microRNA 235, 808, 1168 microsatellite instability 1906 migration 1270, 1575 mineral wool 1797 minimisation 1711 miR-584 308 *miR-1* 808 *miR-133a* 808 miR-148a 1770 miR-34b/c 1770 miRNA 830, 893 missing covariates 693 mitochondria 629 mitochondrial DNA 1319 mitomycin C 1098 mitosis 1523 molecular detection 1178 molecular targeted therapy 941 monoclonal antibody 1106 monoHER 437 mortality 60, 934 motility 308, 1038 mouse model 1452 MRI 578, 1116, 1854 mRNA 819 MRP2 expression 707 mTOR 101, 643, 741 MUC16 989 mucins 989 multidisciplinary clinic 1246 multidrug resistance protein 2 707 multimer technology 948 multiple imputation 693 multivariate analysis 790 mutations 1319 myeloma 281

nasopharyngeal carcinoma 1160 natural killer cells 1106 NC-6004 593 NDPK 1628 neo-adjuvant chemotherapy 707 neovascular- and cancer cell-targeting photodynamic therapy 1401 neuregulin 1135 neuroendocrine tumour 1067 neutropaenia 1377 neutropenic sepsis 407 neutrophil/lymphocyte ratio 1288 NF-*κ*B 166, 1564 NICE guidance 1810 ninein-like protein 1523 nitric oxide 83 Nlp 1523 nodal involvement 714 non-compliance 685 non-Hodgkin's lymphoma 1862 non-melanoma skin cancer 1334 non-small cell lung cancer 413, 1594, 1877 non-small cell lung tumour cells 1410 nonsmokers 208 normal tissue effects 1365 normalisation 1168 North East Netherlands 1193 NSAIDs 763 NSCLC 68, 316 nuclear factor-*κ*B 437 nuclear receptor 448 nuclear sites 12 nucleosomal DNA 719 nucleotides 1628

obesity 51, 875 occupation 208 occupational dust exposure 1797 oesophageal cancer 265, 427 oesophageal cancer family history 841 oesophageal squamous cell carcinoma 707, 841 oestrogen 338 oestrogen receptor 1828 oligodendroglioma 1747 oncogene mutation 464 oncologists 1535 oncolysis 290 OPCML 1313 oral cavity 850 oral squamous cell carcinoma 460, 830 oropharyngeal cancer 886 osteoclastogenesis 505 osteopontin 1007 oval cells 24 ovarian ascites 1602 ovarian cancer 863, 989, 1196, 1241 oxaliplatin 43, 769

p16^INK4a^ 1334 p53 91, 1013, 1278 paclitaxel 1564 pain 1704 palliative care 1704 pancreas 1296 pancreatic cancer 769, 1027, 1038, 1440, 1882 pancreatic cancer-gene expression in 514 panitumumab 1848 Pap-test 248 PARP 750 partner avoidance 1249 partner support 1249 pathologic complete response 1840 patient recruitment 1529 PD 0332991 1862 PD173074 75 pemetrexed 1594 pepsinogens 1511 peripheral blood lymphocytes 1724 peripheral venous blood 1472 persistence 1558 persistent infection 290 personalised screening 1656 personalised therapy 545 pharmacodynamic 756 pharmacoepidemiology 188, 1558 pharmacokinetic 756 phase I study 593, 756 phospholipase 338 phosphorylation 1755 photodynamic therapy 798, 1106 physical activity 882 physical contact 505 PI3K–Akt signalling pathway 146 PI3K-AKT-mTOR 392 PML-RAR*α* 554 poly(A) cDNA 514 polygenic risk 1656 polymer micelle 593 polymorphism 1126 population mixing 12 population-based screening programme 685 postoperative early relapse 1178 post-traumatic stress symptoms 419 prediction 1544 predictive factor 599 preoperative chemoradiotherapy 427 prescription 1704 prevalence 927 primary breast cancer 1393 primary head and neck cancer 1246 primary health care 934 primary systemic therapy 1730 progesterone receptor 921 prognosis 545, 707, 714, 921, 1013, 1168, 1288, 1296, 1418, 1440, 1641, 1680, 1724, 1747, 1762, 1882, 1890, 1913 prognostic factors 564 prognostic markers 856, 893, 1434, 1611, 1619 prolactin receptor 1641 proliferation 345, 1641 prospective studies 419, 524, 1196, 1487 prostate cancer 175, 505, 613, 629, 664, 875, 1656, 1920 prostate epithelial cell lines 673 prostate-specific antigen 613 protein kinase B 1755 protein kinase C, phospholipase D 673 PROX1 1747 proximal tumours 763 psychology 578 PTEN 146, 1587 PTSD 419 PU.1 (SPI1) 554 pulmonary mestastasectomy 1085 punch biopsies 255 purinergic receptors 1628 pyrosequencing 735

quality of life 587, 1697 quality-adjusted survival 1848 quantitative PCR 1342, 1913 quantitative RT–PCR 514

R132H 968 Rab proteins 33 Rab11-FIP 33 radiation 1214 radiation therapy 1365, 1840 radiography 1482 radiotherapy 1724, 1810 Raf 1920 Raf kinases 229 raltitrexed 1869 randomised clinical trial 1816 Ras pathway 229 RAS-RAF-MEK-ERK 392 Rassf polypeptides 24 reactive oxygen species 1564 real-time PCR 332, 1303, 1739 recruitment 1535 recurrence 1762 reduced toxicity 290 registration 1227 relative survival 564 renal cancer 741, 1151 renal cell carcinoma 241, 308, 399, 537, 643, 941, 1256, 1487, 1797 renal transplantation 1334 replication 369 replication inhibitor 1098 resistance 750, 1270 retinoblastoma-positive solid tumours 1862 Rho proteins and Cdc42 324 RhoA 781 risk 181, 1202 risk assessment 1762 Risk of Ovarian Malignancy Algorithm 863 ROCK-1 308 RT–PCR 514 RXR 554

S-1 1126 safety 413, 1691 salt intake 198 SCCHN 1691 Scotland 60 screening 248, 910, 915, 1214, 1680, 1779 SDF-1 1805 second cancer 178 selection 361 selective serotonin reuptake inhibitors 188 self-sampling 915 senescence 700 sensitivity 1049 sensor 1649 sequential therapy 399 serum marker 1418 SGK 1116 side population 1410 sigma1 290 signalling 664, 1628 single-cell PCR 464 single-nucleotide polymorphisms 893 siRNA 1027 skin 1459 SLE 1478 Smad3 33 small molecule inhibitors 653 smoking 198, 1500 Snail 1160 snoRNA 1168 social class 520 social support 1249 socioeconomic circumstance 1791 soft tissue sarcoma 437, 1544 sorafenib 941, 1256 SOX2 1410 squamous cell carcinoma 850, 1106 squamous cell carcinoma of the head and neck (HNSCC) 138 squamous cervical cancer 110 stomach cancer 1193 stomach neoplasms 198 subcellular localisation 841 sunitinib 241, 741, 1686 sunitinib malate 399 sunlight 214 surgery 1393, 1810, 1840 survival 60, 726, 1246, 1680, 1747, 1890 survivin 781, 941 survivorship 927 systematic review 1196 systemic lupus 1478

TACE 138 TAGLN2 808 tamoxifen 899, 1558 targeted therapy 399, 464, 1256, 1686 taxanes 316 temozolomide 1816, 1854 temsirolimus 643 testosterone 1452 tetraspanin 496 tetraspanin 8 155 tetraspanin CD151 1611 TFII-I 1349 *TGFBR1* 735 Thiothymidine 1869 thymidine kinase 332 thymidine phosphorylase 1185 thymidine phosphorylase inhibitor 1185 thymidylate synthase 1594 thyroid atrophy 241 thyroid neoplasms 181 TIMP3 138 Tip-1 324 tissue factor 1401 tissue micro-arrays 693 TKI-258 75 toll-like receptor 2 460 tongue cancer 1890 toxicity 756, 1365 TRAIL 281, 1278 TRAMP 629 transcription factor 1410 transendothelial migration 469 transplantation 433 Trastuzumab 1675, 1716 treatment decision making 1551 treatment sequencing 605, 620 treatment variation 1551 tricyclic antidepressants 188, 193 triple negative 120 *TSP1* 316 tumour angiogenesis 51 tumour endothelial cells 819 tumour growth 166, 496 tumour markers 1342, 1755 tumour morphology 903 tumour neovasculature 1106 tumour regrowth 1805 tumour response 1854 tumour site 198 tumour suppressor pathway 24 tumour thickness 982 tumour vaccine 643 tumour-associated antigen 300 tumourigenesis 1523 twin study 520 *TXR1* 316 type 1 1505 type 2 1505 type II 1611 tyrosine kinase inhibitor 75, 1144, 1686 UICC stage II/III 1178 urothelial carcinoma 75 UVA 1869 uveal melanoma 1098

vaccine 1670 vascular 1877 vasculogenesis 1805 vatalanib 1686 vegetables 6 VEGF 128, 741, 1262, 1270 VEGF-A 819 VEGFR2 128 vesicle trafficking 33 vimentin 1296 vinorelbine 1071 vitamin D 214 volatile biomarker 1649 V-raf murine sarcoma viral oncogene homologue B1 464

waist-to-hip ratio 1207 waiting lists 934 Warburg effect 345 weight 746 Wnt 1906

xenograft 941 XIAP 1575 *XRCC1* 1356

zinc-*α*2-glycoprotein 441

